# Clinical Course of COVID-19 in Children With Adrenal Insufficiency: Results From National Data

**DOI:** 10.1210/clinem/dgaf076

**Published:** 2025-02-07

**Authors:** Donatella Capalbo, Cristina Moracas, Laura Guazzarotti, Federico Baronio, Marianna Rita Stancampiano, Rita Ortolano, Mariella Valenzise, Carla Bizzarri, Giuseppa Patti, Silvia Longhi, Claudia Giavoli, Chiara Guzzetti, Silvia Zoletto, Crescenza Lattanzio, Paolo Cavarzere, Maria Elisabeth Street, Maria Felicia Faienza, Anna Grandone, Marco Cappa, Malgorzata Gabriela Wasniewska, Gianni Russo, Mohamad Maghnie, Mariacarolina Salerno

**Affiliations:** Unit of Pediatric Endocrinology, Department of Medical and Translational Sciences, University of Naples Federico II, 80131 Naples, Italy; Unit of Pediatrics, Department of Mother and Child, University Hospital of Naples Federico II, 80131 Naples, Italy; Pediatric Endocrinology Unit, Department of Woman and Child Health University of Padova, 35128 Padova, Italy; Department Hospital of Woman and Child, Pediatric Unit, IRCCS University Hospital of Bologna, 40138 Bologna, Italy; Department of Pediatrics, Endocrine Unit, IRCCS San Raffaele Scientific Institute, 20132 Milan, Italy; Department Hospital of Woman and Child, Pediatric Unit, IRCCS University Hospital of Bologna, 40138 Bologna, Italy; Department of Human Pathology of Adulthood and Childhood, University of Messina, 98125 Messina, Italy; Unit of Pediatrics, Department of Mother and Child, University Hospital, 98125 Messina, Italy; Unit of Pediatric Endocrinology, Bambino Gesù Children's Hospital, IRCCS, 00165 Rome, Italy; Department of Neuroscience, Rehabilitation, Ophthalmology, Genetics, Maternal and Child Health, University of Genoa, 16100 Genoa, Italy; Department of Pediatrics, Endocrine Unit, IRCCS Istituto Giannina Gaslini, 16100 Genova, Italy; Department of Pediatrics, Hospital of Bolzano (SABES-ASDAA) Teaching Hospital of Paracelsus Medical University, 39100 Bolzano, Italy; Endocrinology Unit, Fondazione IRCCS Ca’ Granda Ospedale Maggiore Policlinico, 20122 Milan, Italy; Department of Clinical Sciences and Community Health, University of Milan, 20122 Milan, Italy; Unit of Pediatric Endocrinology and Neonatal Screening Centre, Microcitemico Pediatric Hospital, 09121 Cagliari, Italy; Pediatric Endocrinology Unit, Department of Woman and Child Health University of Padova, 35128 Padova, Italy; Pediatric Unit, Department of Precision and Regenerative Medicine and Ionian Area, University of Bari “Aldo Moro,” 70124 Bari, Italy; Pediatric Clinic, Department of Surgical Sciences, Dentistry, Gynecology and Pediatrics, University of Verona, 37100 Verona, Italy; Department of Medicine and Surgery, University of Parma, 43126 Parma, Italy; Department of Mother and Child, AUSL-IRCCS of Reggio Emilia, 42122 Reggio Emilia, Italy; Pediatric Unit, Department of Precision and Regenerative Medicine and Ionian Area, University of Bari “Aldo Moro,” 70124 Bari, Italy; Department of Child, Woman, General and Specialized Surgery University of Campania Luigi Vanvitelli, 80138 Naples, Italy; Research Unit Innovative Therapies in Endocrinology, Bambino Gesù Children's Hospital, IRCCS, 00165 Rome, Italy; Department of Human Pathology of Adulthood and Childhood, University of Messina, 98125 Messina, Italy; Unit of Pediatrics, Department of Mother and Child, University Hospital, 98125 Messina, Italy; Department of Pediatrics, Endocrine Unit, IRCCS San Raffaele Scientific Institute, 20132 Milan, Italy; Department of Neuroscience, Rehabilitation, Ophthalmology, Genetics, Maternal and Child Health, University of Genoa, 16100 Genoa, Italy; Department of Pediatrics, Endocrine Unit, IRCCS Istituto Giannina Gaslini, 16100 Genova, Italy; Unit of Pediatric Endocrinology, Department of Medical and Translational Sciences, University of Naples Federico II, 80131 Naples, Italy

**Keywords:** adrenal insufficiency, SARS-CoV-2, COVID-19, pandemic, adrenal crisis

## Abstract

**Context:**

There has been concern about a potential increase in the incidence or severity of coronavirus disease 2019 (COVID-19) in individuals with adrenal insufficiency (AI). Data on the course of SARS-CoV-2 infection in AI children are lacking.

**Objective:**

Evaluate whether children with AI are more susceptible to the infection or are at risk of severe COVID-19.

**Methods:**

In this multicenter, retrospective study among 1143 children with AI, 148 contracted SARS-CoV-2 (112 with primary, 36 with secondary AI) and were evaluated for severity and outcomes of infection, along with 74 control subjects with normal adrenal function.

**Results:**

The prevalence of COVID-19 in the AI cohort was 12.9%, not increased compared to pediatric Italian population in the same period. The severity was not increased in AI subjects and was classified as follows in patients vs controls: asymptomatic in 14.9% vs 10.8%; paucisymptomatic in 33.8% vs 37.8%; mild in 45.3% vs 45.9%; severe in 3.4% vs 2.7%; critical in 2.7% vs 2.7%. Among those with severe COVID, 4 patients with AI (2.7%) and 3 controls (4%) developed pneumonia while 3 patients with PAI (2%) and 2 controls (2.7%) developed multisystem inflammatory syndrome (*P* not statistically significant). Only 5 patients (3.4%) experienced an adrenal crisis during a severe COVID-19. The hospitalization rate was the same in patients vs controls (9.5%). All subjects completely recovered, and no COVID-related deaths were documented.

**Conclusion:**

Our findings do not indicate that AI is associated with increased susceptibility to SARS-CoV-2 infection or higher risk for severe COVID-19 in children.

Coronavirus disease 2019 (COVID-19), caused by the severe acute respiratory syndrome coronavirus 2 (SARS-CoV-2), led to a global pandemic (1, 2). In general, children tend to experience mild cases of COVID-19. The rate of hospitalization among children with COVID-19 varies from 0.1% to 10%, depending on the severity of the illness and the population studied (3, 4) and mortality is exceedingly rare (5, 6). However, a small proportion of children is at risk of severe illness and of developing multisystem inflammatory syndrome (MIS-C) (7). Risk factors for a more severe outcome include underlying medical conditions, although there is still limited evidence linking specific conditions with unfavorable outcomes (8, 9). Adrenal insufficiency (AI) is a rare condition caused by insufficient cortisol production (10). AI can be primary (PAI), resulting from adrenal gland failure, or secondary (SAI) and tertiary AI, arising from pituitary gland defects or the exogenous use of steroids, respectively (10). Hydrocortisone is the primary glucocorticoid (GC) used for replacement therapy in children with AI (10, 11).

Patients with AI face increased overall morbidity and mortality (12, 13). They are particularly vulnerable to infections (14-16), during which they are at increased risk of developing serious complications, especially life-threatening adrenal crisis (AC). Mechanisms behind the increased susceptibility to infections in individuals with PAI remain unclear. Recent research has suggested that impaired natural killer cell function may be a contributing factor (17). Additionally, since glucocorticoids can both stimulate and suppress the immune response, variations in GC doses or treatment regimens could influence this effect (15, 18). In this context, concerns have arisen regarding the potential for an increased incidence or severity of COVID-19 in individuals with AI (19). While there is no conclusive evidence of increased severity in these patients (20, 21), some studies have suggested higher odds of contracting COVID-19 or increased hospitalization rates in adults with AI (22-25). However, many of these studies were based on data from national registries, lacked case-control analyses, and were heterogeneous in study design. Additionally, they included few pediatric patients, leaving limited evidence on the impact of SARS-CoV-2 infection in children with AI. The objective of our multicenter study is to assess the effect of AI and GC replacement therapy on the prevalence and outcomes of SARS-CoV-2 infection in a large cohort of pediatric patients diagnosed with AI.

## Methods

### Study Population

Fourteen Italian centers participated in a multicenter retrospective study involving children with AI who contracted COVID-19 between March 2020 and May 2022.

AI was diagnosed based on low basal or stimulated cortisol levels, associated with increased adrenocorticotropic hormone (ACTH) in patients with PAI or low/low-normal ACTH in patients with SAI (26). In subjects with congenital adrenal hyperplasia (CAH) due to 21-hydroxylase deficiency (21-OHD) or to other defects, the diagnosis relied on concentration of basal or stimulated 17-hydroxyprogesterone (17-OHP) and/or other adrenal precursors (27).

Subjects with tertiary AI related to exogenous administration of steroids were excluded. All patients with adjunctive risk factors for severity of COVID-19, such as severe obesity (body mass index [BMI] > 3 SDS or >99.9th percentile) (28), heart disease, pulmonary disease, hypertension, active malignancy, or immunodeficiency, were excluded.

**Figure 1.**
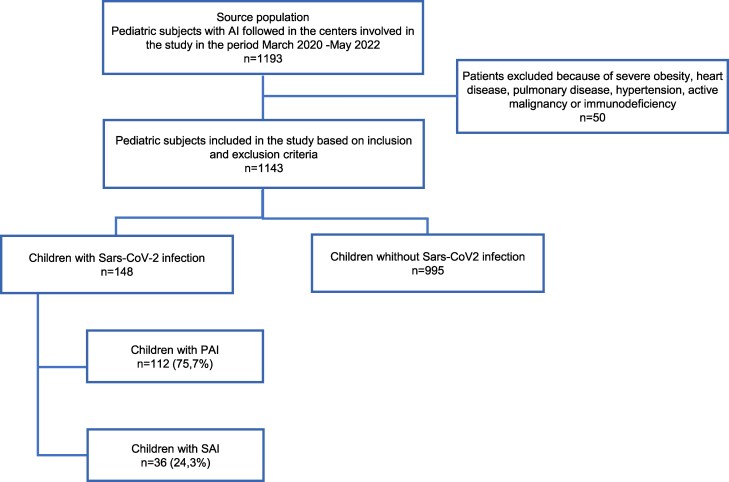
Overview of the recruitment of children with adrenal insufficiency who contracted COVID-19 between March 2020 and May 2022.

Based on the above inclusion and exclusion criteria, 1143 subjects with AI were identified from a total cohort of 1193; among these subjects, 148 contracted SARS-CoV-2 between March 2020 and May 2022 and were included in the study: 112 (75.7%) had a diagnosis of PAI and 36 (24.3%) a diagnosis of SAI (Fig. 1).

**Figure 2.**
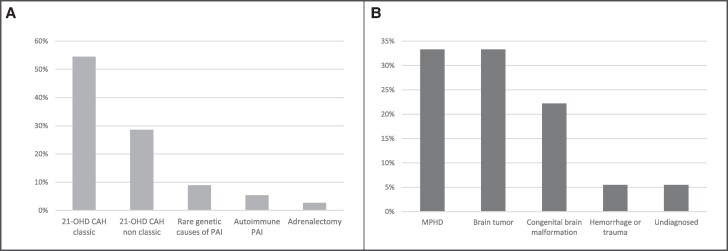
Etiologies in patients with PAI (A) and SAI (B) who experienced COVID-19 between March 2020 and May 2022.

In patients with PAI, the mean age at diagnosis of AI was 3.3 ± 4.5 years; 61/112 subjects (54.5%) had a diagnosis of classic 21-OHD; 32/112 (28.6%) had non-classic 21-OHD; 10/112 (8.9%) had other conditions such as 11beta-hydroxylase deficiency, X-linked adrenoleukodystrophy, adrenal hypoplasia congenita, or familial glucocorticoid deficiency; 6/112 (5.4%) had autoimmune AI and 3/112 patients (2.7%) developed PAI after adrenalectomy (Fig. 2A).

In patients with SAI, the mean age of 6.5 ± 4.8 years at diagnosis of AI was significantly higher than those with PAI (*P* < .001); 12/36 subjects (33.3%) had idiopathic multiple pituitary hormone deficiencies; 12/36 subjects (33.3%) developed SAI after brain tumors; 8/36 (22.2%) had congenital brain malformations; 2/36 subjects (5.5%) developed SAI following brain hemorrhage and trauma, respectively; in 2 subjects the etiology of SAI was not identified (Fig. 2B). Among them, 34/36 had multiple pituitary hormonal deficiencies. In detail, 28 had thyrotropin (thyroid stimulating hormone) deficiency, 26 had growth hormone deficiency, 12 had luteinizing hormone/follicle stimulating hormone deficiency, and 11 had central diabetes insipidus.

Seventy-four subjects (M/F = 38/36), of comparable age and sex to the patients, who contracted COVID-19 in the same period of AI subjects were enrolled as controls. Of these control subjects, 44 (59.5%) were healthy children referred to outpatient clinic for family history of thyroid disease or growth assessment; 30 (40.5%) were followed for other chronic endocrinopathies that did not compromise adrenal function: 7 were followed for premature telarche, 12 for congenital hypothyroidism, and 11 for isolated growth hormone deficiency, all adequately replaced (Supplementary Table S1) (29). As for patients, severe obesity and concomitant adjunctive risk factors for COVID-19 infection severity were considered exclusion criteria.

### Study Design

Data regarding the clinical course of COVID-19 were retrospectively retrieved from patient records and missing information was collected by telephone. In all subjects, COVID-19 was confirmed by real-time reverse-transcriptase polymerase chain reaction of SARS-CoV-2 RNA in nasopharyngeal swab specimens or viral antigens using a rapid antigen detection test. SARS-CoV-2 variants have been hypothesized based on the date of the positive test: infections that occurred before December 2021 were considered as Alpha, Beta, and Delta variants and those after December 2021 as Omicron variants.

### Data Collection and Outcomes

To define the clinical course of COVID-19, the following data were collected in all subjects: date and age of onset; signs, symptoms, and duration of the infection; treatments; complications; and hospital admissions. Laboratory tests were recorded when available.

The severity of COVID-19 was defined based on the clinical presentation: (i) asymptomatic: no apparent symptoms of disease; (ii) paucisymptomatic: dry cough, general malaise, headache, low-grade fever, tiredness; (iii) mild: uncomplicated upper respiratory tract viral infection and/or gastrointestinal symptoms (eg, vomiting, diarrhea, abdominal pain) with or without fever; (iv) severe: pneumonia, hypoxia dyspnea, tachypnea, or severe dehydration with electrolyte abnormalities; (v) critical: severe pneumonia, acute respiratory distress syndrome, septic shock, MIS-C, and/or multiple organ dysfunction requiring intensive care (30). The duration of COVID-19 was considered as the time between the first positive swab and the first negative one.

In subjects with AI, data on current treatment, on the control of underlying condition, and on adherence to therapy were collected. The control of underlying disease was defined as “good” or “poor” based on clinicians’ judgments and biochemical markers prior to infection. Information about administration of stress or parenteral doses of hydrocortisone and occurrence of AC during COVID were collected. AC was defined as an acute deterioration in health status associated with hypotension, nausea/vomiting, or severe fatigue, or electrolyte abnormalities that resolve after parenteral glucocorticoid administration (31). Information on vaccination status were collected for each subject.

This study was approved by the Ethical Committee of the coordinating center (No. 259/21). Informed consent was obtained.

### Statistical Analysis

Statistical analysis was performed using the software SPSS (SPSS Statistics for Windows, version 26.0, IBM Corp). Continuous quantitative variables are expressed as mean ± SD and categorical variables are expressed as frequency distribution percentage (%). Comparison of variables was performed using the Student *t* test or the chi-square test.

Correlation analysis was used to evaluate those variables associated with the outcome of COVID-19 in pediatric patients with AI to investigate independent factors associated with the course of infection.

The level of significance was set at 0.05.

## Results

Based on the total cohort of 1143 subjects with AI involved in the study, the overall incidence of COVID-19 in our selected pediatric population with AI was 12.9% (Fig. 1).

Details of 148 patients with AI and 74 controls who experienced SARS-CoV-2 infection are reported in Table 1.

**Table 1.** Details of patients with adrenal insufficiency and controls who contracted COVID-19 between March 2020 and May 2022

	AI	Controls	*P* value*^a^*	PAI	SAI	*P* value*^b^*
Number	148	74	NA	112	36	NA
Male/Female	77/71	38/36	NS	58/54	19/17	NS
Age at AI diagnosis (years)	4.1 ± 4.8	NA	NA	3.3 ± 4.5	6.5 ± 4.8	<.001
Age at COVID-19 (years)	11.2 ± 4.8	10.3 ± 4.5	NS	10.8 ± 4.7	12.4 ± 4.9	NS
Body mass index	20.4 ± 3.9	19.5 ± 3.7	NS	20.2 ± 3.9	20.8 ± 3.7	NS
Puberty, No. (%)	87 (58.7)	47 (63.5)	NS	67 (59.8)	20 (55.5)	NS
HC dose (mg/m^2^/d)	12.6 ± 4.6	NA	NA	13.8 ± 4.5	9.0 ± 2.6	<.001
FC dose (mg/d)	0.1 ± 0.04	NA	NA	0.1 ± 0.04	NA	NA
Duration of COVID-19 symptoms (days)	4.9 ± 6.4	6.4 ± 7.4	NS	5.3 ± 7.0	4.2 ± 6.0	NS
Duration of COVID-19 infection (days)	14.4 ± 8.0	14.1 ± 7.8	NS	14.6 ± 8.5	13.8 ± 6.5	NS
Increase oral HC during COVID-19, No. (%)	87 (58.8)	NA	NA	63 (56.3)	24 (66.7)	NS
Parenteral HC during COVID-19, No. (%)	9 (6.1)	NA	NA	7 (6.3)	2 (5.6)	NS
Adrenal crisis during COVID-19 No. (%)	5 (3.4)	NA	NA	4 (3.6)	1 (2.8)	NS

Abbreviations: AI, adrenal insufficiency; BMI, body mass index; FC, fludrocortisone; HC, hydrocortisone; NA, not applicable; NS, not significant; PAI, primary adrenal insufficiency; SAI, secondary adrenal insufficiency.

^a^ *P* value AI patients vs controls.

^b^ *P* value PAI vs SAI.

No differences in age at COVID infection, BMI, nor pubertal status were found between AI and controls nor between PAI and SAI (Table 1). Moreover, we did not find significant differences in BMI, pubertal development, and treatment doses between COVID-positive patients and a subgroup of COVID-negative PAI and SAI for whom data were available (Supplementary Table S2) (29).

**Figure 3.**
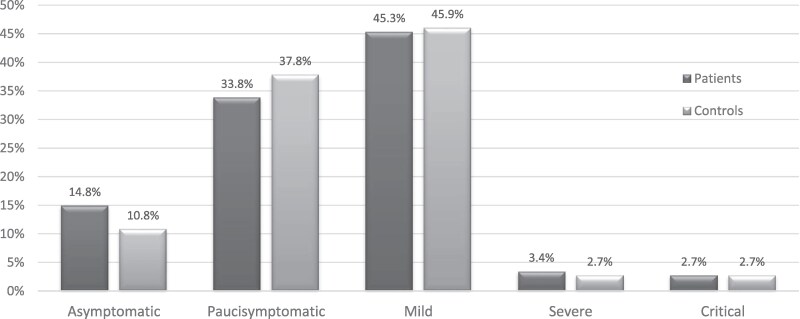
Severity of COVID-19 in patients with adrenal insufficiency compared to controls.

The severity and the type and frequency of signs and symptoms of COVID-19 were not significantly different in AI subjects compared to controls and most subjects developed paucisymptomatic or mild disease (Fig. 3).

**Figure 4.**
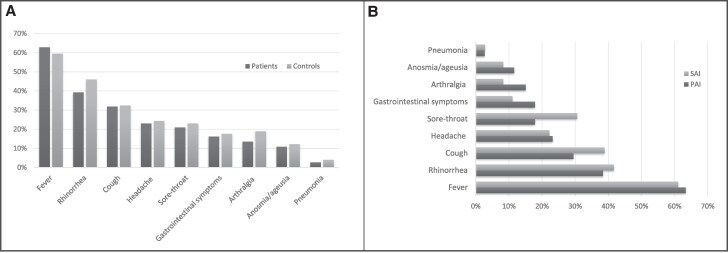
Signs and symptoms of COVID-19 in children with adrenal insufficiency vs controls (A) and in PAI vs SAI (B).

Fever was the most common sign in patients and controls (62.8% vs 59.5%) followed by cough (31.8% vs 32.4%) and rhinorrhea (39.2% vs 46.0%). Sore throat, headache, and arthralgia had also similar frequency in patients (20.9%, 23%, and 13.5%) and controls (23.0%, 24.3%, and 18.9%). Vomiting, abdominal pain, and diarrhea were reported in 16.2% of patients and 17.6% of controls. Anosmia and/or ageusia were reported by 10.8% of patients and 12.2% of controls. 4 patients with AI (2.7%), 3 with PAI, and 1 with SAI, and 3 controls (4%) developed pneumonia (*P* = not significant) (Fig. 4A). MIS-C developed in 3 patients with AI (2%), and 2 healthy controls (2.7%).

No significant difference was found in signs and symptoms between males and females nor between patients with PAI or SAI (Fig. 4B).

Overall, the same percentage of patients (n = 14, 9.5%) and controls (n = 7, 9.5%) was hospitalized. However, in 5 patients and 3 controls the reasons for hospitalization were not related to the severity of COVID-19 but rather to other factors such as age, or increased caution of parents and physicians. None of them required intensive care and all subjects fully recovered.

The duration of COVID-19 did not differ between patients and controls nor between PAI and SAI subjects (Table 1). None of the patients with AI received antiviral therapy during SARS-CoV-2 infection.

When evaluating the distribution of symptoms based on the presumptive variant of SARS-CoV-2, we documented that gastrointestinal symptoms occurred more before December 2021 (*P* = .006) while anosmia and ageusia were not detected after December 2021. A trend toward milder symptoms was observed in children who contracted the infection after December 2021, although this was not significant.

Among the patients with AI, 58% increased their daily glucocorticoid dosage during the infection following sick-day rules; only 5 patients (3.4%), 4 with PAI and 1 with SAI, developed an AC during a severe or critical course of COVID-19.

No significant correlation was detected between severity of SARS-CoV-2 infection and the dose of hydrocortisone (*P* = .120), dose of fludrocortisone (*P* = .847), and control of the underlying disease (*P* = .940) No differences in the severity of COVID were observed between AI subjects with normal weight (70.9%) and those with overweight (19.6%) and obesity (9.5%) as well between overweight/obese PAI and SAI patients (Supplementary Table S3) (29).

Finally, only 15/148 subjects with AI (10%), 9 with PAI and 6 with SAI, had received at least one dose of SARS-CoV-2 vaccination before the infection occurred. In this group of patients, no cases of severe COVID-19 were recorded and the duration of infection was shorter than in unvaccinated (10.1 ± 4.4 vs 16.0 ± 8.5 days, *P* = .01).

## Discussion

Our findings do not indicate an increased susceptibility to SARS-CoV-2 infection or a higher risk of severe COVID-19 in children with AI.

The COVID-19 pandemic has been a period of great uncertainty for individuals with AI, given their vulnerability to infections, which significantly contribute to their overall morbidity.

Since the onset of COVID-19, few studies have focused on the disease's impact in AI patients. Currently, there is no clear evidence suggesting a higher prevalence or severity of COVID-19 in this population (19), although data remain inconclusive. In 2020, a retrospective study involving 279 adults with AI and 112 controls reported no differences in the incidence or severity of infection between patients and controls (20). Similarly, a recent study of 54 CAH patients found no increased prevalence of SARS-CoV-2 infection or more severe outcomes in patients compared to the general population (21). Conversely, an analysis of European Registries for Rare Endocrine conditions, which included 57 AI patients, found a high rate of hospitalization (16%) among adult patients older than 37 years, attributed to AC or severe infections (32). In line with these findings, a study of 5430 adults with AI from Swedish national registries observed an infection rate similar to controls but a higher incidence of hospitalization, intensive care needs, and death in AI patients (22). Additionally, a multinational study reported an increased relative risk of infection and hospitalization in subjects with CAH, particularly those over 40 years of age, males, those with pulmonary disease, or those on higher maintenance glucocorticoid doses (23).

These studies have several limitations. First, many included small, unselected samples of AI patients without control groups. Second, SARS-CoV-2 infection was often only suspected rather than consistently confirmed through laboratory tests. Third, much of the data came from national registries rather than individual patient evaluations. Additionally, only a small number of children were included in the analyses, leading to insufficient data to assess the impact of SARS-CoV-2 infection in children with AI.

Our study does not indicate that AI or GC treatment increases susceptibility to SARS-CoV-2 infection. While the incidence of COVID-19 in children might be underestimated due to the high rate of asymptomatic cases, we found a 12.9% incidence in pediatric AI patients, lower than the 37.4% infection rate among Italian children during the same study period (33, 34). Furthermore, children with AI in our experience had mild, self-limiting courses of SARS-CoV-2 infection, with the frequency of severe or critical cases being similar to controls and consistent with other pediatric studies (around 7%) (3, 35, 36). Hospitalization rates between patients and controls were also comparable. Although the rate was at the higher end of reported ranges (3, 4) it is important to note that some hospitalizations may have been due to younger age or increased caution from doctors and parents regarding the underlying condition, potentially overestimating the number of AI children requiring hospital care for COVID-19.

Among AI patients who experienced severe COVID-19, we were unable to identify risk factors related to underlying etiology, treatment, or control of adrenal disease. Unlike reports on adults suggesting that patients with SAI may be more severely affected than those with PAI (22), we have documented no differences in the incidence or severity of infection between children with PAI or SAI; furthermore, no gender-related differences were detected. Weight excess seemed to not significantly impact on the susceptibility to infection nor on its severity in AI children since the course of COVID was comparable between normal weight and overweight/obese patients; however, we did not investigate the outcome in severe obese patients.

While concerns exist about depleted immunity in subjects with AI, the relatively mild course of COVID-19 suggests that their antiviral immune defense is not significantly compromised. However, studies are needed to evaluate immune function in patients with AI.

SARS-CoV-2 did not act as a significant trigger for AC in our patients, as its frequency was not elevated compared to that reported in pediatric populations with AI (37-39). Adrenal crisis events occurred only in patients with severe or critical COVID-19, and, as expected, those with PAI were more prone to develop AC than those with SAI (38). Notably, no COVID-19-related deaths were recorded among patients or controls. One important factor that may explain the differences between our findings and adult studies, which report a more severe course of infection, is the higher prevalence of comorbidities in adults with chronic AI patients, making them more vulnerable to severe COVID-19 outcomes compared to children. Additionally, other factors may have contributed to the mild course of infection in children with AI. It is possible that children with AI and their families adopted especially strict social distancing and personal protection measures during the pandemic and were more likely to use stress doses to prevent AC. Lastly, vaccination likely influenced the course of the disease (40), with vaccinated patients experiencing a shorter illness duration than nonvaccinated patients. However, the number of vaccinated subjects was too small to draw definitive conclusions about the protection of the vaccines against severe disease.

Our study has several strengths, including the involvement of a large, carefully selected cohort of pediatric patients with AI, assessed at the individual level. The study also featured a control group, laboratory confirmation of SARS-CoV-2 infection, detailed information on treatments, the use of stress doses, and data on the occurrence of adrenal crises. By excluding patients with preexisting comorbidities, we eliminated potential confounding factors, enabling a clearer evaluation of the direct impact of adrenal dysfunction and chronic steroid use on disease progression. However, we acknowledge that the exclusion of AI subjects with severe obesity and other comorbidities may represent a limitation since it could have been interesting to evaluate if children with these features and AI have more severe COVID-19 the those without risk factors. In particular, the role of obesity should be further investigated in larger samples of subjects with different degrees of weight excess. Another main limitation of the study is its retrospective design and the characteristics of the control group, which included both healthy children and those with underlying endocrine disorders not affecting adrenal function. However, all control subjects with endocrine conditions were in good health and well-managed, meaning that they did not represent a high-risk population.

## Conclusions

Our findings are reassuring and indicate that neither AI nor glucocorticoid treatment is linked to an increased susceptibility to SARS-CoV-2 infection or a higher risk of severe COVID-19 in children. Importantly, our results highlight that proper and continuous education on adhering to sick-day rules is crucial for ensuring safe clinical outcomes during illness. These data suggest that children with AI, appropriately treated and monitored, maintain an effective first-line defense against viral infections, offering valuable insights into managing concerns about compromised innate immunity in these patients.

## Disclosures

Authors have nothing to disclose.

## Data Availability

The datasets for this article are available on reasonable requests, which should be directed to the corresponding author (Mariacarolina Salerno). Any data sharing will be subject to meeting the Regulations of Local Ethical Committee.

## Abbreviations

21-OHD: 21-hydroxylase deficiency
17-OHP: 17-hydroxyprogesterone
AC: adrenal crisis
AI: adrenal insufficiency
BMI: body mass index
CAH: congenital adrenal hyperplasia
GC: glucocorticoid
MIS-C: multisystem inflammatory syndrome
PAI: primary adrenal insufficiency
SAI: secondary adrenal insufficiency
